# Persistent infection with *Porphyromonas gingivalis* increases the tumorigenic potential of human immortalised oral epithelial cells through ZFP36 inhibition

**DOI:** 10.1111/cpr.13609

**Published:** 2024-02-13

**Authors:** Ze Lu, Ruoyan Cao, Fengxue Geng, Yaping Pan

**Affiliations:** ^1^ Department of Periodontics, School of Stomatology China Medical University Shenyang China

## Abstract

The association between *Porphyromonas gingivalis* infection and oral squamous cell carcinoma (OSCC) has been established by numerous epidemiological studies. However, the underlying mechanism specific to this connection remains unclear. By bioinformatical analysis, we identified ZFP36 as a potentially significant co‐expressed gene in both the OSCC gene database and the persistent infection model of *P. gingivalis*. To further investigate the role of ZFP36, we established a cell model that human immortalized oral epithelial cells (HIOECs) that were sustainedly infected by *P. gingivalis* (MOI = 1) for a duration of 30 weeks. Our findings indicated that sustained infection with *P. gingivalis* inhibited the expression of ZFP36 protein and induced changes in the biological behaviour of HIOECs. The mechanism investigation demonstrated the potential role of ZFP36 in regulating the cancer‐related biological behaviour of HIOECs. Subsequent studies revealed that highly expressed CCAT1 could serve as a molecular scaffold in the formation of the ZFP36/CCAT1/MK2 complex. This complex formation enhanced the binding abundance of MK2 and ZFP36, thereby promoting the inhibition of ZFP36 protein phosphorylation. To summarize, low expression of ZFP36 protein under persistent *P. gingivalis* infection enhances the cancer‐related biological behaviour of HIOECs.

## Graphical abstract

Persistent infection with *Porphyromonas gingivalis* can inhibit ZFP36 by forming ZFP36/CCAT1/MK2 complexes and enhance the tumorigenic potential of human‐immortalised oral epithelial cells.

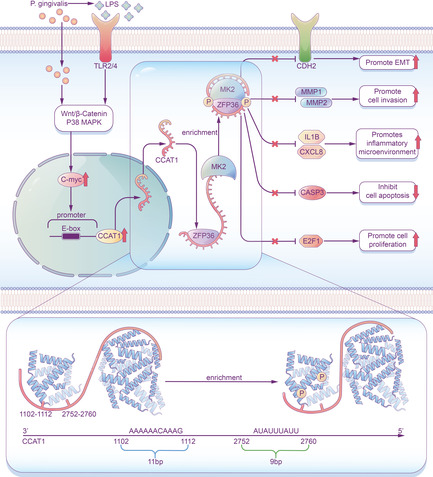

## 1 INTRODUCTION

Periodontitis is an infectious disease initiated by dental plaque, which is characterised by progressive destruction of periodontal supporting tissues (gingiva, periodontal ligament, alveolar bone and cementum) and eventual loss of teeth.^1^ *Porphyromonas gingivalis* is a gram‐negative anaerobic bacterium which is considered one of the key pathogens of periodontitis. It is classified within the ‘red complex’ alongside *Tannerella forsythia* and *Treponema denticola*.^2^ The main virulence factors of *P. gingivalis* include lipopolysaccharide (LPS), gingipain, fimbriae outer membrane vesicles (OMVs), and so forth,^3^ The main pathogenic mechanism of *P. gingivalis* is believed to primarily induce inflammatory reactions and destroy or evade the innate immune defence mechanisms of the human body.^4^ *P. gingivalis* is categorised as a late colonising bacterium within the dental plaque biofilm, facilitating the establishment of a suitable environment for other biofilm constituents through adhesion. Furthermore, *P. gingivalis* exhibits the ability to enhance the maturity and stability of the biofilm by modulating the metabolites present within it. Additionally, *P. gingivalis* collaborates with other biofilm components to evade both innate and adaptive immune responses, thereby promoting the advancement of periodontitis.^5^

These years, there has been a growing interest in the correlation between *P. gingivalis* and oral squamous cell carcinoma (OSCC).^6^ While the conventional belief linked OSCC to tobacco and alcohol consumption, recent studies suggest that specific constituents of the oral microbiome also contribute to the development of OSCC.^7^, ^8^ A research conducted on clinical samples revealed that individuals diagnosed with OSCC exhibited elevated levels of *P. gingivalis* in their gingival tissue compared to healthy individuals.^9^ Furthermore, a comprehensive review and meta‐analysis examining the relationship between *P. gingivalis* and oral cancer indicated that the presence of *P. gingivalis* was associated with a 1.36‐fold (95% CI = 0.47–3.97) increased risk of developing cancer.^10^ These findings suggest that *P. gingivalis* may play a significant role in the development of OSCC.

*P. gingivalis* can be recognised by Toll‐like receptors 4 (TLR4),^11^ leading to the activation of various pro‐inflammatory cytokines through the STAT and NFκB signalling pathways. The release of pro‐inflammatory cytokines creates a conducive inflammatory environment for the initiation and progression of cancer.^12^ Furthermore, the immunosuppressive capabilities of *P. gingivalis* also play a significant role.^13^, ^14^, ^15^ For instance, *P. gingivalis* was found to be able to suppress the host's adaptive immune system by manipulating immune cells, especially T cells and B cells.^16^, ^17^ However, whether there is a deeper link between the inflammatory environment induced by *P. gingivalis* infection and the cancer‐promoting phenotype remains unanswered.

ZFP36 (also known as tristetraprolin, TTP) belongs to the zinc finger protein family. It is a regulator of many proinflammatory proteins, including its own.^18^ ZFP36 is considered to be a global regulator of inflammatory response.^19^ Mice with ZFP36 knockout exhibited severe systemic inflammation and autoimmune symptoms.^20^ ZFP36 functions primarily as an RNA binding protein (RBP) for mRNA decay by binding to the 3′‐untranslated regions (UTRs) of AU‐rich element mRNA (ARE‐mRNA).^21^, ^22^ In the last decade, more than 20 types of cancer were found to be linked to ZFP36.^23^ Due to the fact that ZFP36 targets genes involved in tumorigenesis and progression, its downregulation in human cancer is associated with poor prognosis.^24^ ZFP36 is involved in the regulation of a variety of biological behaviours of tumour cells, including uncontrolled cellular proliferation in the absence of external growth signals, resistance to apoptosis, sustained angiogenesis, as well as tissue invasion and metastasis.^23^ The role of ZFP36 in OSCC has not been fully explored, but it has been linked to head and neck squamous cell carcinoma (HNSCC). The down‐regulation of ZFP36 can significantly enhance the invasion of tumour cells into the basement membrane by activating IL‐6 and MMPs in HNSCC.^25^ High levels of ZFP36 can also enhance the sensitivity of tumour cells to cisplatin by inhibiting BCL‐2, an anti‐apoptotic protein.^26^ Nevertheless, it is worthwhile to explore whether the dysregulation of ZFP36 plays a potentially significant role in the process of OSCC formation by modulating the inflammatory microenvironment.

The stability of ARE–mRNA is heavily regulated by the P38 mitogen‐activated protein kinase (MAPK) pathway.^27^ P38 is activated by bacterial LPS, TNF‐α and other pro‐inflammatory stimuli and then phosphorylates substrates including MAPK‐activated protein kinase 2 (MAPKAPK2, MK2).^28^ MK2 is the primary protein recognised for controlling ZFP36. MK2 phosphorylates Ser‐52 and Ser‐178 sites in mice and Ser‐186 in humans of ZFP36, creating a substrate for adaptor protein 14‐3‐3,^29^ which protects ZFP36 from dephosphorylation of protein phosphatase 2A (PP2A).^30^ It was reported that phosphorylated ZFP36 lost its ability to bind to ARE‐mRNA and thus stabilised mRNA.^31^ Knocking out MK2 in mice resulted in a significant decrease in the levels of TNF‐α, IL‐1, IL‐6 and IFN‐γ.^32^ Despite this, there is also evidence that ZFP36 retains its affinity for its target gene but cannot function under P38 activation conditions.^33^ This may be related to the inability of phosphorylated ZFP36 to locate processing bodies (PBs). While we currently know that the stable regulation of ARE‐mRNA is mediated by both P38 and ZFP36, the detailed underlying mechanisms require further investigation.

Long non‐coding RNAs (LncRNA) is a class of non‐coding RNAs with more than 200 nucleotides in length that regulate gene expression at different levels such as chromatin, splicing, transcription and post‐transcriptional levels, which is involved in a variety of biological processes, such as cell proliferation, invasion, metastasis and apoptosis.^34^ Among all lncRNAs, colon cancer‐associated transcription‐1 (CCAT1), also known as cancer‐associated region long non‐coding RNA‐5 (CARR‐5) has received increasing attention. Studies showed that CCAT1 performed a high‐expression pattern and carcinogenic effect in different types of tumours and abnormal expression of CCAT1 is involved in tumour occurrence, progression, metastasis and patient survival by regulating different target genes and signalling pathways.^35^ CCAT1 currently has four common action modes. First, CCAT1 forms a chromatin ring by binding to the CCCT‐binding factor (CTCF), acting as an RNA‐binding protein.^34^ Second, CCAT1 forms DNA/RNA/protein complexes that regulate protein enhancer activity.^36^ Third, CCAT1 can serve as a scaffold for two epigenetic modification complexes.^37^ Last, CCAT1 acts as a competitive endogenous RNA (ceRNA).^38^ However, considering the diversity of lncRNA regulatory mechanisms, the functional role of CCAT1 and its mechanism in tumorigenesis promoted by bacterial infection remain to be determined.

In this study, we aimed to explore the potential link between persistent infection with *P. gingivalis* and the abnormal biological behaviour of human immortalized oral epithelial cells (HIOECs). The possible key genes for OSCC were identified by comparing the genetic data of patients with OSCC in the TCGA public database with the genetic data of the *P. gingivalis* persistent infection cell model. The expression of target genes was verified using clinical samples and a model of *P. gingivalis* persistent infection with HIOECs was established to explore the possible mechanism.

## 2 RESULTS

### 2.1 Inflammation is strongly associated with OSCC based on bioinformatical analysis

**FIGURE 1:**
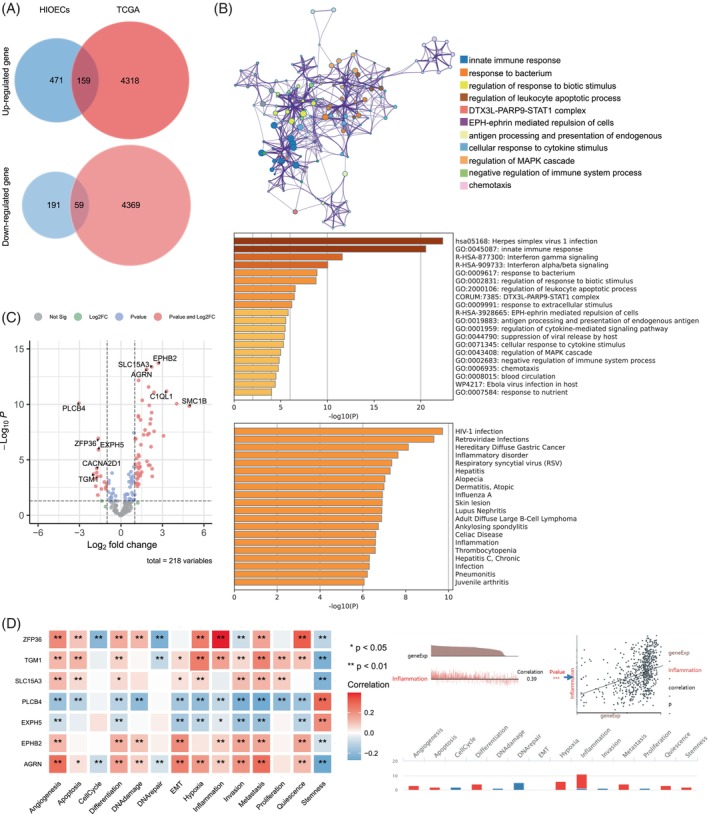
Bioinformatics analysis. (A) oral squamous cell carcinoma grouping data from the TCGA database and HIOECs chip data from *Porphyromonas gingivalis* persistent infection were co‐expressed by gene Venn diagram, 218 co‐expressed genes were found, among which 159 co‐expressed genes had increased expression and 59 co‐expressed genes had decreased expression. (B) GO analysis showed that co‐expressed genes were associated with exogenous microbial infection and inflammatory processes. (C) Volcano map of TOP10 coexpressed genes. The top 10 genes were *PLCB4*, *ZFP36*, *EXPH5*, *CACNA2D1*, *TGM1*, *EPHB2*, *SLC15A3*, *AGRN*, *C1QL1*, and *SMC1B*. (D) Correlation analysis diagram of TOP10 genes and tumour‐related biological behaviours. ZFP36 was strongly associated with the inflammatory phenotype (*p < 0.05, **p < 0.01).

To investigate the significant association between persistent infection of *P. gingivalis* and the development of OSCC, we acquired the genetic information of patients eith OSCC from the publicly available TCGA database. Through a comparative analysis of the TCGA gene data and the microarray data obtained from our previously established model that HIOECs were persistently infected by *P. gingivalis*, we identified a total of 218 co‐expressed genes, with 159 genes exhibiting up‐regulation and 59 genes displaying down‐regulation (Figure 1A). Enrichment analysis by cell type indicated that the co‐expressed genes exhibited associations with various epithelial cell types, such as tongue epithelial cells and lung epithelial cells (Figure S1A). The gene ontology (GO) analysis further indicated a strong correlation between the co‐expressed genes and exogenous microbial infection as well as inflammatory processes (Figure 1B). A comprehensive overview of the GO analysis results can be found in Figure S1B. Analysis of transcription factors also indicated that co‐expressed genes were strongly linked to the transcription factors associated with inflammatory pathways, such as STAT1 and NFκB (Figure S1C).

### 2.2 ZFP36 is chosen as a key gene associated with OSCC and inflammation

The volcano map illustrating co‐expressed genes was constructed based on the statistical significance of expression differences. The analysis revealed that the top 10 genes, namely *PLCB4*, *ZFP36*, *EXPH5*, *CACNA2D1*, *TGM1*, *EPHB2*, *SLC15A3*, *AGRN*, *C1QL1* and *SMC1B* (Figure 1C). Subsequent analysis of the relationship between these genes and tumour‐related biological behaviours, including angiogenesis, apoptosis, cell cycle, differentiation, DNA damage, DNA repair, EMT, hypoxia, inflammation, invasion, metastasis, proliferation, quiescence and stemness, demonstrates that *PLCB4*, *ZFP36*, *EXPH5*, *TGM1*, *EPHB2*, *SLC15A3* and *AGRN* exhibited associations with tumour‐related biological behaviours. Specifically, *ZFP36* displayed the most significant association with the inflammatory response (Figure 1D). Moreover, the analysis of tumour grade showed that lower *ZFP36* expression was associated with higher tumour grade (Figure S1D). Consequently, *ZFP36* was chosen as the focal gene for this study.

### 2.3 The expression of ZFP36 is reduced in the clinical samples of periodontitis and OSCC

**FIGURE 2:**
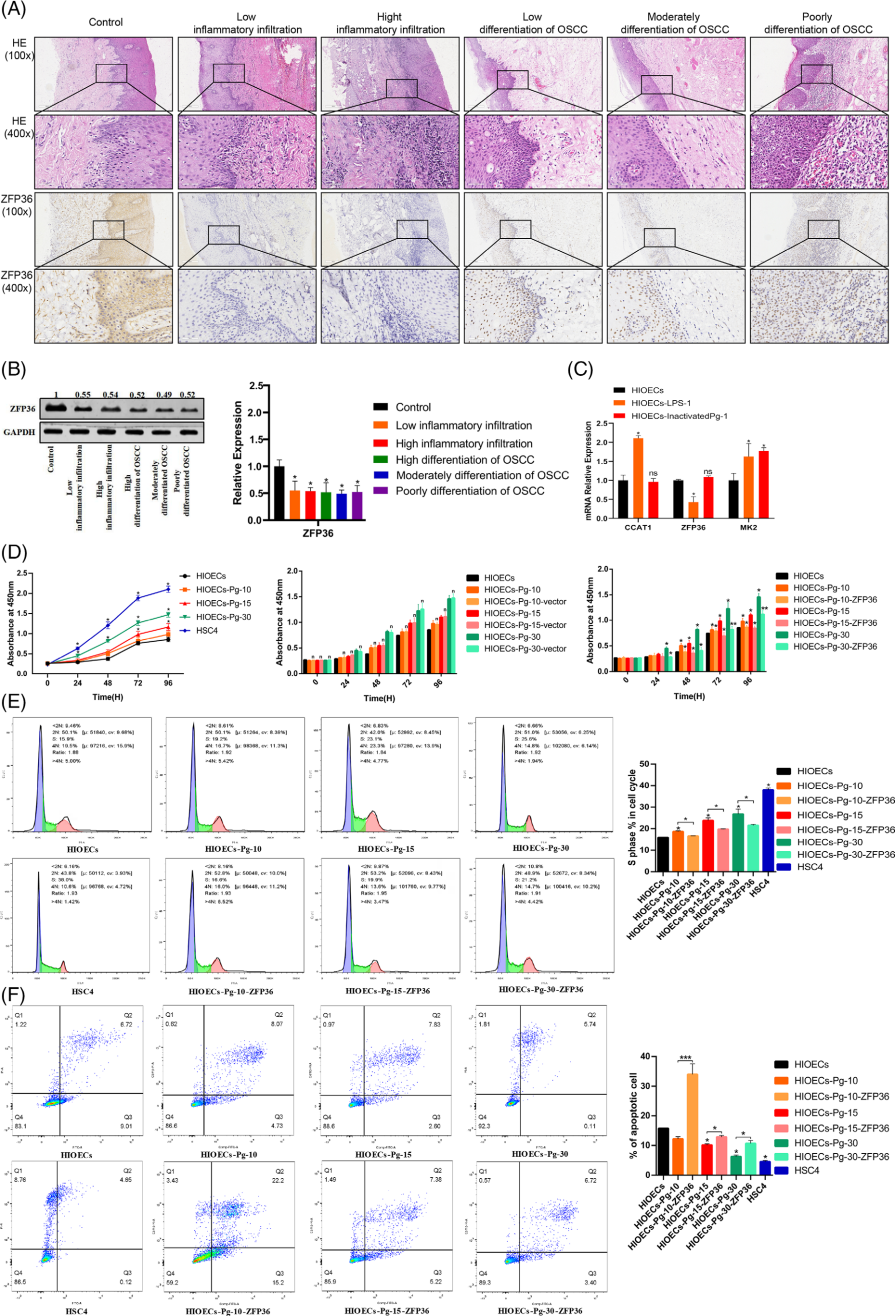
Clinical sample validation. (A) HE staining and immunohistochemical staining results of ZFP36 proteins in each group of periodontitis and oral squamous cell carcinoma samples. The results of ZFP36 staining showed that the expression of ZFP36 in tissues decreased gradually with the aggravation of inflammation. (B) Western blot results of ZFP36 and content in tissue samples. The number above the black band represents the relative expression of the grey value. (C) Expression of CCAT1, ZFP36, MK2 genes in LPS and inactivated *Porphyromonas gingivalis* for 1 week (*p < 0.05, **p < 0.01). (D) Results of cell proliferation detection in *P. gingivalis* persistent infection model. The proliferative ability of infected cells was gradually increased (*p < 0.05, **p < 0.01). (E) Cell cycle test results of *P. gingivalis* persistent infection model, The percentage of S‐phase cells increased gradually (*p < 0.05, **p < 0.01). (F) Results of apoptosis detection in *P. gingivalis* persistent infection model. The apoptosis ability of infected cells was gradually decreased (*p < 0.05, **p < 0.01).

Clinical samples of gingival epithelial tissue were collected from patients diagnosed with periodontitis, while cancerous tissue samples were obtained from patients with OSCC. To ensure comparability, factors such as gender, age and systemic disease were balanced. Monocyte chemoattractant protein‐1 (MCP‐1) staining was employed to assess the level of inflammation in all tissue samples, including those from the healthy control group, low inflammation group and high inflammation group (Figure S2A). The findings from ZFP36 staining indicated a gradual decrease in ZFP36 expression in tissues as inflammation worsened. This trend in ZFP36 expression is also evident in the western blot analysis of tissue samples (Figure 2A,B). It is noteworthy to mention that the level of inflammation observed in OSCC tissues is comparable to that observed in the low‐inflammation group of periodontitis tissues. Conversely, the high‐inflammation group exhibits significant damage to tissue cells, characterised by a substantial infiltration of inflammatory cells, oedema and necrosis of epithelial tissue (Figure 2A). The findings indicate that inflammation in cancerous tissue appears to be regulated within a specific range and excessive inflammation can rapidly damage the tissue.

### 2.4 *P. gingivalis* persistent infection promoted a variety of cancer‐related biological behaviours in HIOECs

**FIGURE 3:**
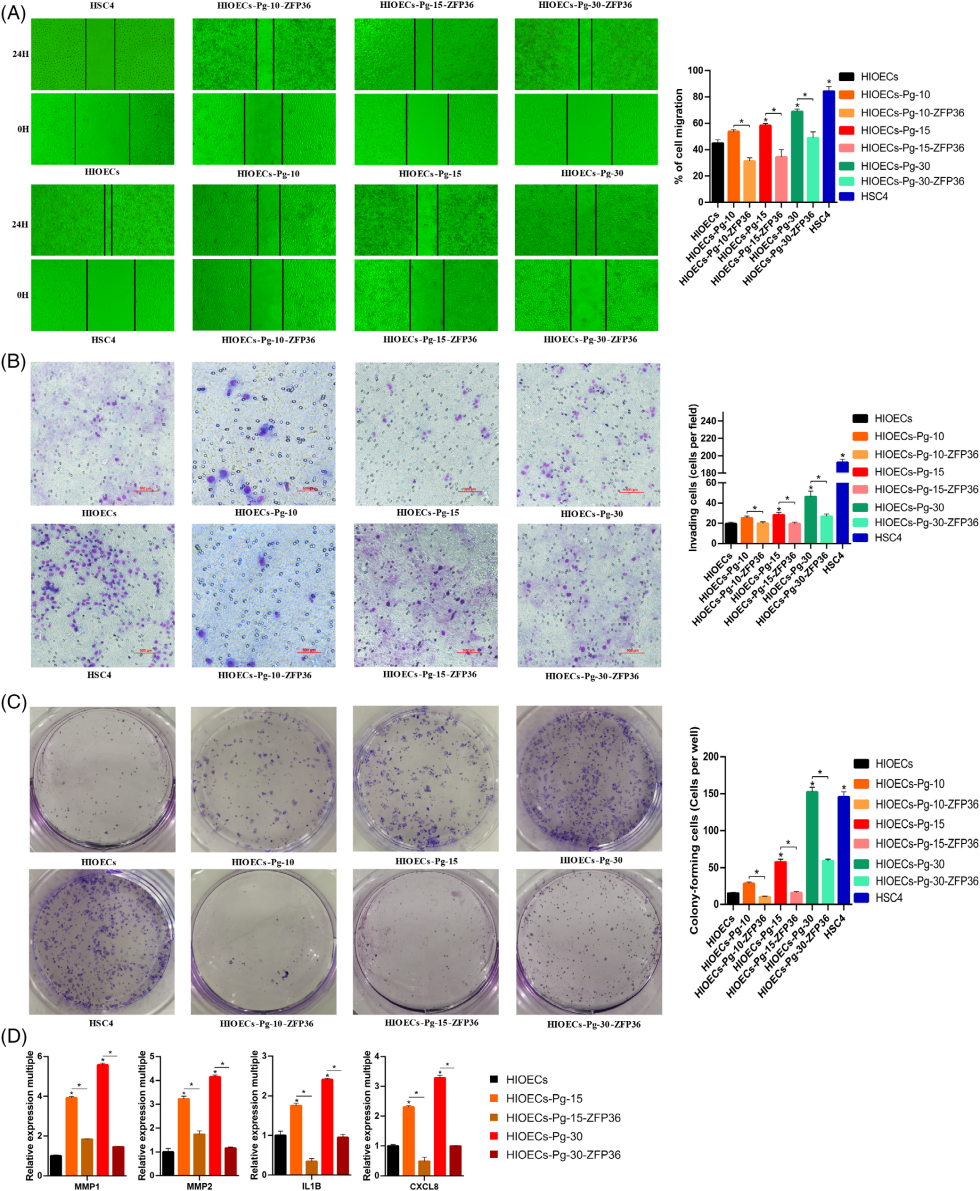
Results of the tumour‐associated biological phenotype. (A) Results of cell migration detection in *Porphyromonas gingivalis* persistent infection model. The migration ability of infected cells was gradually increased (*p < 0.05, **p < 0.01). (B) Results of cell invasion detection in *P. gingivalis* persistent infection model. The invasion ability of infected cells was gradually increased (*p < 0.05, **p < 0.01). (C) Detection results of colony formation ability in *P. gingivalis* persistent infection model. The colony formation ability of infected cells was gradually increased (*p < 0.05, **p < 0.01). (D) Detection results of secretory cancer‐promoting factor directly regulated by ZFP36. MMP1, MMP2, IL1B and CXCL8 were significantly elevated (*p < 0.05, **p < 0.01).

In vitro, we established an infection model using HIOECs that were infected with a low dose of *P. gingivalis* (multiplicity of infection = 1, MOI = 1) for a duration of 30 weeks. Cell biological behaviours in HIOECs cells infected with *P. gingivalis* for durations of 10, 15 and 30 weeks were examined, with human tongue squamous cell carcinoma cell line (HSC4) serving as the positive control. The findings indicated a gradual increase in the proliferative ability of infected cells (Figure 2D). Furthermore, there was a gradual increase in the percentage of S‐phase cells (Figure 2E), accompanied by a gradual decrease in the overall apoptosis rate. Notably, the apoptotic distribution of HIOECs‐Pg‐30 cells closely resembled that of HSC4 cells (Figure 2F). Additionally, the migration, invasion and colony formation capacities of the cells exhibited a gradual enhancement (Figure 3A–C). ELISA was employed to identify secreted factors linked to cancer promotion that were under direct regulation by ZFP36.^25^, ^39^, ^40^ The findings revealed a noteworthy increase in the expression levels of MMP1, MMP2, IL1B and CXCL8 (Figure 3D).

### 2.5 Overexpression of ZFP36 can improve the biological behaviour of HIOECs induced by *P. gingivalis* infection

**FIGURE 4:**
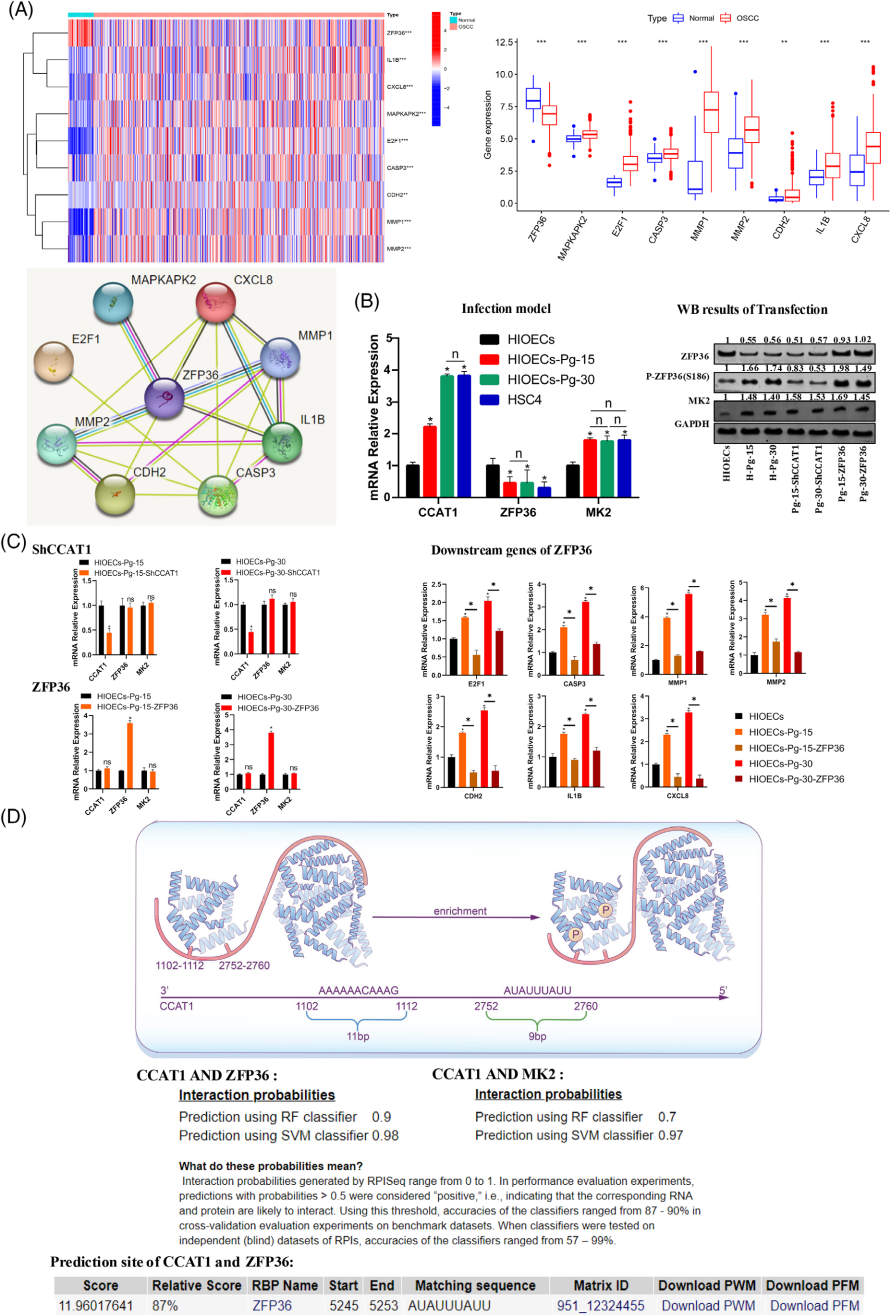
Screening results of genes and proteins interacting with ZFP36. (A) The TCGA database screened the genes and proteins directly interacting with ZFP36 upstream and downstream (*p < 0.05, **p < 0.01, ***p < 0.001). (B) Expression results of ZFP36, CCAT1, MK2 genes and proteins in a 30‐week *Porphyromonas gingivalis* persistent infection model. The expression of CCAT1 gradually increased with the increase of *P. gingivalis* infection time. MK2 gradually increases during weeks 0 to 10 but remains unchanged and reaches HSC4 levels during weeks 10 to 30. The WB results show that P‐ZFP36 (S186) was progressively elevated from 0 to 30 weeks compared to the control group. MK2 protein expression gradually increased during weeks 0 to 10 and remained unchanged during weeks 10 to 30. After inhibition of CCAT1, the expression of P‐ZFP36 (S186) was gradually decreased. After overexpression of ZFP36, the expression of P‐ZFP36 (S186) gradually increased. However, the expression of MK2 was not affected (*p < 0.05). (C) The changes of related genes after inhibiting CCAT1 and overexpressing ZFP36, respectively (*p < 0.05). (D) The combined interaction of CCAT1 with ZFP36 and MK2 predicted the results respectively.

As the duration of *P. gingivalis* infection increased, the mRNA expression of ZFP36 exhibited a gradual decrease from 0 to 15 weeks, followed by a stable level from 15 to 30 weeks (Figure 4B). Conversely, the total protein levels of ZFP36 gradually decreased throughout the entire 30‐week period (Figure 4B). To further investigate the involvement of ZFP36 in tumour‐related biological processes, ZFP36 was overexpressed using a transfection plasmid. The findings demonstrated significant inhibition of tumour‐related biological behaviour in the cells following ZFP36 overexpression (Figures 2D–F and 3A–C). Moreover, the downregulation of downstream pro‐oncogenes and inflammatory cytokines, which are directly regulated by ZFP36, was observed to be decreased (Figure 4C). These findings indicate that the continuous presence of *P. gingivalis* infection has the potential to inhibit the expression of ZFP36. Furthermore, the overexpression of ZFP36 can improve the effects of *P. gingivalis* infection on cell biological behaviour and certain pro‐inflammatory cytokines.

### 2.6 CCAT1 regulates the levels of ZFP36 protein and is associated with the phosphorylation of ZFP36

To investigate the precise mechanism underlying the down‐regulation of ZFP36 protein, we employed an online database to predict the molecules involved in the regulation of ZFP36. Furthermore, we searched for these genes in the microarray data from the previous cellular model.^41^ Our attention was drawn to CCAT1 and MK2 proteins due to their significant relevance. Specifically, CCAT1, characterised as an oncogene, exhibits multiple binding sites with ZFP36 and displays abnormally high expression levels under *P. gingivalis* stimulation. The protein MK2, which is extensively investigated, plays a crucial role in the regulation of ZFP36 and was demonstrated to exhibit binding affinity towards ZFP36 (Figure 4A). Furthermore, the potential binding interaction involving CCAT1, ZFP36 and MK2 was predicted. The results indicated a substantial likelihood of CCAT1 binding with both ZFP36 and MK2 (Figure 4D).

**FIGURE 5:**
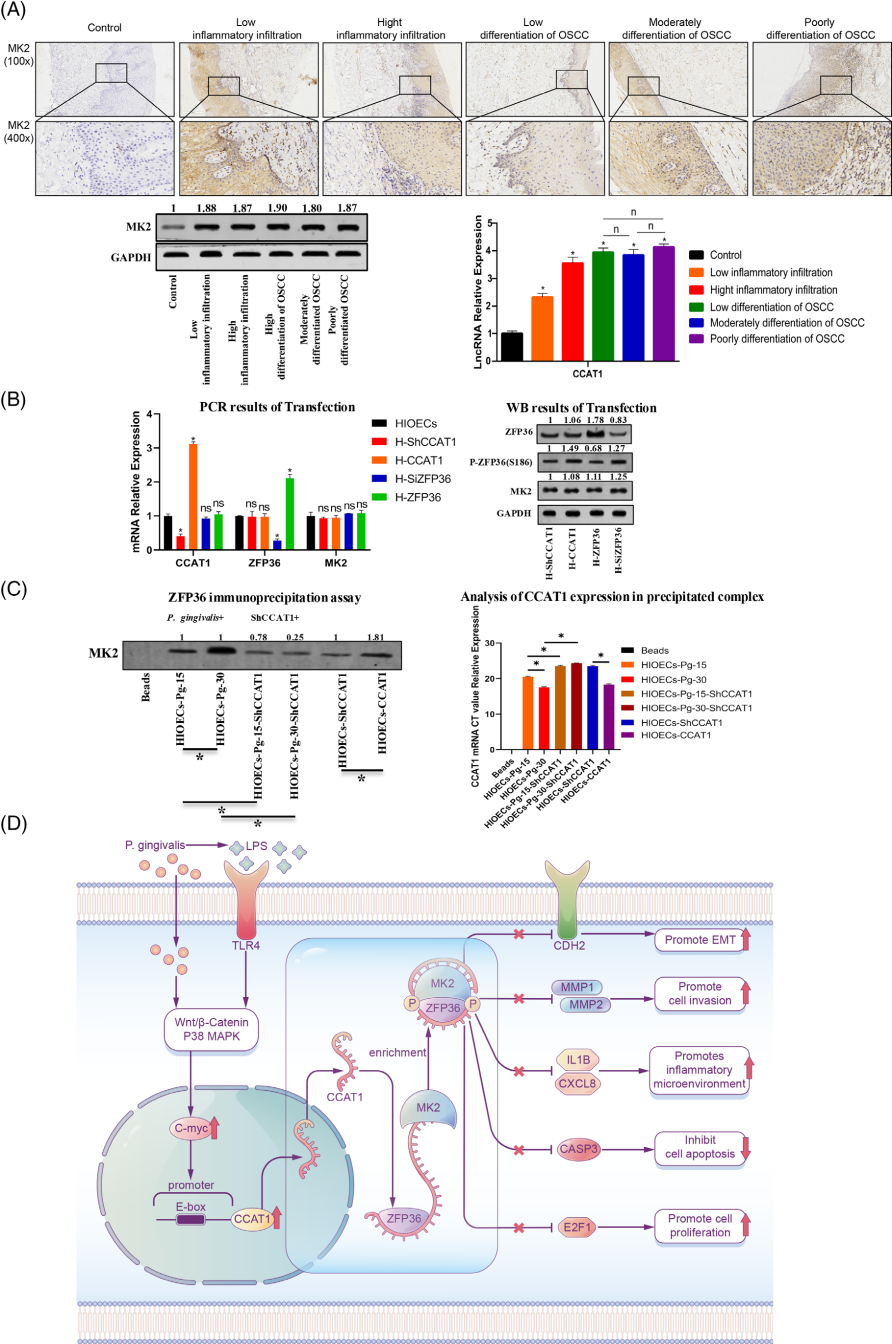
Verification results of the binding relationship between CCAT1 and MK2 expression levels and ZFP36/CCAT1/MK2 complex. (A) The expression of CCAT1 and MK2 was verified in clinical samples. The expression of MK2 in clinical tissue samples was detected. The expression of CCAT1 in periodontitis and OSCC tissues was gradually increased, while the expression of MK2 was unchanged (*p < 0.05). (B) RIP experimental cell samples were constructed (*p < 0.05, **p < 0.01). (C) RIP verifies the structure of the ZFP36/CCAT1/MK2 complex. When the ZFP36 protein was pulled, CCAT1 and MK2 proteins were detected in the precipitated complex, suggesting that the three proteins combined to form a complex. When the expression of CCAT1 was changed, the content of MK2 protein in the precipitate complex increased with the increase of CCTA1 (*p < 0.05, **p < 0.01). (D) The mechanism diagram of this study.

To verify the potential interactions among CCAT1, ZFP36 and MK2, The expression of CCAT1 and MK2 was first examined in clinical tissue samples. The findings indicated a progressive increase in the expression of CCAT1 in periodontitis and OSCC tissues compared to healthy controls, while the expression of MK2 remained unchanged (Figure 5A). Subsequently, the qPCR results obtained from the cell model demonstrated a gradual up‐regulation of CCAT1 expression with prolonged *P. gingivalis* infection. Additionally, the expression of MK2 exhibited a gradual increase within the initial 15 weeks. However, between the 15th and 30th weeks, the expression of MK2 remained constant and was comparable to that observed in HSC4 cells (Figure 4B). When the expression of CCAT1 was suppressed through transfection with a transfection plasmid, the expression of ZFP36 and MK2 remained unaltered (Figure 4C). However, the mRNA expression of ZFP36 did not exhibit any changes during the 15‐ to 30‐week period, while the protein level gradually decreased, suggesting potential post‐translational modification of ZFP36. Therefore, the protein expression of p‐ZFP36 at S186 was assessed. The expression of p‐ZFP36 (S186) exhibited a progressive increase from 0 to 30 weeks in comparison to the control group (Figure 4B). Conversely, the protein expression of MK2 demonstrated a gradual increase during weeks 0–15, followed by a stable level from weeks 15–30 (Figure 4B). Upon inhibition of CCAT1, the expression of p‐ZFP36 (S186) displayed a gradual decrease (Figure 4B). Conversely, upon overexpression of ZFP36, the expression of p‐ZFP36 (S186) exhibited a gradual increase, while the expression of MK2 remained unaffected (Figure 4B). These results suggest that ZFP36 is gradually phosphorylated and deactivated during *P. gingivalis* persistent infection, which is mainly regulated by CCAT1 and is related to the phosphorylation of ZFP36 by MK2 at the S186 site.

### 2.7 CCAT1 inhibits ZFP36 protein by forming the ZFP36/CCAT1/MK2 complex

To further investigate the specific role of CCAT1 in facilitating the phosphorylation of ZFP36, we conducted RNA‐binding protein immunoprecipitation (RIP) experiments using cell models. By utilizing ZFP36 as the tagged protein, we were able to identify the presence of CCAT1 and MK2 proteins in the precipitated complex, suggesting their association in the formation of the complex of ZFP36/CCAT1/MK2. Notably, upon transfection with overexpression of CCAT1, there was an observed increase in the quantity of MK2 protein within the precipitation complex (Figure 5B,C). This finding supports the prediction from the database that the expression level of CCAT1 influences the binding of MK2 protein to ZFP36. Hence, we postulated that CCAT1 potentially serves as a molecular scaffold, enhancing the binding abundance of MK2 and ZFP36, thereby indirectly facilitating the inhibition of ZFP36 protein phosphorylation by MK2.

## 3 DISCUSSION

These years, specific microbial infections were suggested to exert a significant influence on the process of tumour formation, development and progression.^42^ In the present study, we identified a robust correlation between persistent infection of *P. gingivalis* and OSCC by employing bioinformatics analysis. Among the aberrantly expressed genes, ZFP36 displayed the most significant association with both inflammatory pathways and tumour‐related biological behaviours. We also found that sustained infection of HIOECs by *P. gingivalis* can enhance the expression of CCAT1, potentially implicating it in the regulatory processes underlying OSCC development. Further, CCAT1 was identified to act as a molecular scaffold, facilitating the formation of the ZFP36/CCAT1/MK2 complex. This complex was found to enhance the inhibition of ZFP36 phosphorylation by MK2. Reduced expression of ZFP36 therefore alleviated the inhibition of cancer‐related biological processes, including proliferation, cell cycle regulation, apoptosis, migration and invasion, which consequently improved the tumorigenic potential of HIOECs (Figure 5D).

It was revealed that merely 10% of cancer incidences can be ascribed to germline mutations, while the majority of cancers are instigated by acquired factors. Within this realm of acquired factors, chronic inflammation emerges as a significant contributor to tumorigenesis.^43^ In our bioinformatics analysis, we elucidated the co‐expression gene characteristics of patients with OSCC in conjunction with a persistent infection cell model of *P. gingivalis* (Figure 1A,B). The findings revealed a substantial activation of exogenous bacterial infection and tumour‐promoting inflammatory pathways, thereby corroborating our hypothesis. Subsequent analysis highlighted ZFP36 as one of the 10 genes exhibiting the most significant differences, indicating a strong association with inflammatory processes (Figure 1C,D). ZFP36 was identified as a primary regulator of numerous inflammatory diseases, with its pivotal role initially elucidated through the use of ZFP36 gene knockout mice.^20^ However, the specific involvement of ZFP36 in *P. gingivalis* infection remains largely unexplored. To delve deeper into the role of ZFP36 in *P. gingivalis* infection, we have successfully established a laboratory model utilizing HIOECs, which has allowed for continuous infection for a remarkable duration of 30 weeks, longer than any previously reported infection models, to preserve as many epigenetic alterations as possible. Our findings indicate that the alteration of the ZFP36 protein has an impact on the expression levels of MMP1, MMP2, IL1B and CXCL8 in the supernatant. These genes were previously demonstrated to be directly regulated by ZFP36 in other studies and were associated with the development of a pro‐tumour inflammatory microenvironment.^39^, ^40^, ^44^ Additionally, it is noteworthy that characteristic markers of *P. gingivalis* infection, such as IL6, IL8, MMP9 and HIF‐1, have also been shown to be directly regulated by ZFP36 and contribute to the establishment and maintenance of the inflammatory microenvironment.^25^, ^45^, ^46^ Furthermore, ZFP36 was shown to have an impact on immune cell function in addition to its role in regulating the inflammatory microenvironment. A study conducted by Wang et al. demonstrated that the absence of ZFP36 led to an increase in tumour infiltrating CD8 + T cells in mice, which was attributed to the direct regulation of IL27 by ZFP36.^47^ Similarly, Kratochvill et al. discovered that ZFP36 was consistently highly expressed in tumour‐associated macrophages.^48^ These findings suggest that ZFP36 may also play a role in modulating the inflammatory microenvironment during persistent infection of HIOECs by *P. gingivalis*. It is worth mentioning that we separately examined the LPS of *P. gingivalis* and the effect of heat‐inactivated *P. gingivalis* on HIOECs. The results showed that LPS of *P. gingivalis* could promote the expression of CCAT1 and reduce the transcription level of ZFP36, but heat‐inactivated *P. gingivalis* did not have the above phenomenon. These results suggest that LPS and various enzymes secreted by *P. gingivalis* may play an important role in the activation of CCAT1, but the immune components retained after thermal inactivation such as fimbriae seem to be insufficient to activate CCAT1 expression and the specific mechanism needs to be further studied (Figure 2C).

Carcinogenic microorganisms typically exhibit shared attributes, including persistent colonisation, the generation of chronic inflammatory stimuli, evasion or suppression of immune responses, activation of cancer‐promoting genes or inhibition of cancer‐suppressing genes and the facilitation of epigenetic defect accumulation.^49^ Consequently, infected cells exhibit heightened tumour‐related biological phenotypes, such as increased proliferation, inhibition of apoptosis, enhanced invasion and migration capabilities and augmented colony formation abilities.^49^ Previous research demonstrated that the internalisation process mediated by the cytoskeleton enables *P. gingivalis* infection to infiltrate cells and facilitate intercellular transfer, thereby evading humoral immunity.^50^ Additionally, persistent *P. gingivalis* infection was found to activate well‐established inflammatory pathways associated with cancer promotion, including STAT, NFκB, MAPK and c‐Myc.^51^ Moreover, investigations revealed that *P. gingivalis* persistent infection can impede the functionality of P53, a crucial gene responsible for suppressing tumour development.^52^ While several studies indicated that persistent infection of *P. gingivalis* can enhance tumour‐related biological behaviours, there exists a dearth of consistent regulatory molecules at the mechanistic level. This study posits that ZFP36 might assume this crucial role. We found that changing the expression level of ZFP36 can effectively affect a variety of tumour‐related biological behaviours (Figures 2D–F and 3A–C). The modulation of tumour‐related biological behaviours by ZFP36 is associated with its capacity to bind to and disrupt pivotal regulatory factors. Specifically, ZFP36 was demonstrated to directly regulate key cycle regulators c‐Myc and cyclin D1 in terms of cell proliferation capacity.^53^, ^54^ Additionally, the present study observed that ZFP36 exerted an influence on the expression of the cell cycle checkpoint protein E2F1, a finding that aligns with the findings reported by Lee et al.^55^ E2F1 plays a crucial role in governing the progression from the G1 to the S phase and is frequently overexpressed in various human cancers. Dysregulation of E2F1 expression was linked to the development of malignancy, metastasis and unfavourable prognosis in patients.^55^ Furthermore, with regard to its involvement in apoptosis, ZFP36 was demonstrated to participate in the regulatory mechanisms of apoptosis through direct modulation of the BCL‐2 and TNF‐α pathways.^56^, ^57^ This study also identified the impact of ZFP36 on the expression of caspase3 mRNA. caspase3, a pro‐apoptotic protein, can have its activity enhanced by ZFP36 through the inhibition of human apoptotic protein inhibitor‐2 (cIAP2).^58^ Furthermore, the overexpression of ZFP36 was demonstrated to reverse the epithelial‐mesenchymal transition (EMT) phenotype and directly regulate key regulatory factors such as ZEB1, SOX9, TWIST1 and SNAIL1, which are associated with migration, invasion ability and EMT.^59^ In the present study, we confirmed that the levels of MMP1, MMP2 and CDH2 are influenced by the expression of ZFP36. This finding complements previous research on the migratory and invasive capabilities as well as the EMT. Utilizing the existing model, we examined the impact of ZFP36 on tumour‐associated biological processes (proliferation, cell cycle, apoptosis, migration, invasion, colony formation) and provided evidence that ZFP36 governs the tumour‐related biological behaviour of *P. gingivalis* during persistent infection in HIOECs.

MK2 plays a pivotal role in modulating the functionality of ZFP36, making it the most crucial kinase in this regard. Despite certain investigations revealed the binding of MK2 to ZFP36, several studies failed to regulate the expression of ZFP36 by directly inhibiting MK2.^25^, ^56^, ^58^ This implies that numerous unidentified mechanisms pertaining to the phosphorylation of ZFP36 within the p38 MAPK–MK2 pathway still remain to be elucidated. In this study, our findings indicate that CCAT1 functions as a molecular scaffold, facilitating the binding between MK2 and ZFP36 and subsequently promoting the inhibition of ZFP36 phosphorylation. While the molecular scaffold mode of CCAT1 for two epigenetic modification complexes has been previously described,^38^ its specific mechanism as a molecular scaffold in the context of *P. gingivalis* infection was not explored in previous studies.^38^ We confirmed that *P. gingivalis* infection in HIOECs activates the TLR/P38 MAPK/c‐Myc signalling pathway,^60^ leading to increased expression of CCAT1, which aligns with the findings of Wang et al. (Figure S1B,C). The overexpression of CCAT1 leads to the formation of novel complexes with the ZFP36/MK2 complex. Alterations in the expression level of CCAT1 result in corresponding changes in the MK2 protein content within the complex (Figure 4B), consequently affecting the degree of phosphorylation inhibition of ZFP36 (Figure 4B). These findings contribute to the understanding of the regulatory mechanism of the p38 MAPK–MK2 pathway on ZFP36, indicating that non‐coding RNA may serve as a significant auxiliary regulator in this classical pathway.

In summary, our study provided evidence that prolonged infection of HIOECs with *P. gingivalis* (MOI = 1) leads to a substantial enhancement in the tumorigenic potential of cells. Furthermore, we identified the significance of ZFP36 protein expression in this mechanism. Additionally, our findings reveal a novel observation that highly expressed CCAT1 can form a complex with ZFP36 and MK2, resulting in increased binding abundance between MK2 and ZFP36. This complex formation ultimately facilitates the inhibition of ZFP36 phosphorylation, thereby alleviating the inhibitory effect of ZFP36 on downstream key oncogenes. Simultaneously, the investigation of the specific binding sites of the ZFP36 complex is warranted, considering the multiple phosphorylation sites associated with this protein. The specific roles of *P. gingivalis* components and virulence factors are also worth further exploration. The findings of this study indicate that repeated and persistent *P. gingivalis* infection could potentially contribute to the unfavourable prognosis observed in patients with periodontitis in the context of oral clinical practice. Therefore, stable control of periodontal infection is of great significance for not only oral but also systemic health of patients. This study established a reliable foundation for examining the relationship between persistent *P. gingivalis* infection and OSCC, while also offered a novel approach for managing periodontitis and implementing early prevention and treatment strategies for OSCC.

## 4 MATERIALS AND METHODS

See the Data S1: Appendix for detailed materials and methods.

### 4.1 Bacteria culture

*Porphyromonas gingivals* ATCC 33277 (American Type Culture Collection, Manassas, VA, USA) was routinely cultured. The colonies on the plates were suspended in a TSB liquid medium after 12 h of anaerobic culture and then suspended in phosphate‐buffered saline (PBS, 0.01 M, pH 7.4). Spectrophotometer was used at 600 nm wavelength and adjusted to 1 × 10^9^ CFU/mL (OD = 1). The culture purity of *P. gingivalis* growing in broth was detected by Gram staining and then added into cells according to MOI = 1. *P. gingivalis*‐LPS was obtained from InvivoGen, San Diego, CA, USA and used as per the manufacturer's instructions. When we thermally inactivated *P. gingivalis*, The *P. gingivalis* cell density was adjusted to 1.0 × 10^10^ CFU/mL and the bacterial suspension was heat‐inactivated at 60°C for 1 h and stored at −80°C until use.

### 4.2 Eukaryotic cells culture

Human immortalised oral epithelial cells (HIOECs), a kind of oral epithelial cells collected from patients with cleft lip and palate, were kindly provided by Prof. Wantao Chen from Key Laboratory of Shanghai Oral Medicine, Ninth People's Hospital, Shanghai Jiao Tong University. HIOECs were cultured in Defined Keratinocyte‐SFM (Gibco™, Thermo Fisher Scientific Inc., MA, USA) with growth supplement in a humidified atmosphere at 37°C (5% CO_2_).

Human tongue squamous cell carcinoma (HSCC) cell line HSC4 was purchased from the Japanese Collection of Research Bioresources Cell Bank (JCRB, Japan). HSC4 cells were cultured in 90% Eagle's minimum essential medium (Sigma) supplemented with 10% FBS (Sigma) at 37°C, with 5% CO_2_.

### 4.3 Establishment of long‐term infection model

For long‐term infection, actively growing HIOECs in six‐well plates were infected with *P. gingivalis* at an MOI of 1 for 24 h at each passage. HIOECs were repeatedly infected with *P. gingivalis* for time periods of 30 weeks, named HIOECs‐Pg‐30. HIOECs stimulated continuously for 10 and 15 weeks were selected as intermediate transition indexes, named HIOECs‐Pg‐10 and HIOECs‐Pg‐15, respectively. HIOCEs without *P. gingivalis* challenge as the negative control was sub‐cultured together with infected groups. HSC4 without *P. gingivalis* challenge as the positive control was subcultured together with infected groups.

### 4.4 Cell proliferation (CCK‐8), cell cycle and apoptosis analysis, cell migration (wound healing and transwell) and invasion assays, colony formation experiments

See the Data S1: Appendix.

### 4.5 Immunohistochemical assay, quantitative real‐time polymerase chain reaction, Western Blotting, vectors construction

See the Data S1: Appendix.

### 4.6 Plasmid transfection

See the Data S1: Appendix.

### 4.7 RNA immunoprecipitation (RIP)

Cells were transfected with Inhibition and overexpression plasmids of CCAT1 (Genechem, Shanghai, China). After 48 h, cells were used to perform RIP experiments using a ZFP36 antibody (Cat. no. Sc‐374305; Santa Cruz) and the Magna RIP™ RNA‐Binding Protein Immunoprecipitation Kit (Millipore, Bedford, MA, USA) according to the manufacturer's instructions. Precipitates were purified and analysed by PCR and Western blotting by standard procedures using indicated antibodies at a dilution of 1:500.

### 4.8 Statistical analysis

See the Data S1: Appendix.

## AUTHOR CONTRIBUTIONS

Yaping Pan: Contributed to conception, design, grasp the research direction and critically revised the manuscript. Fengxue Geng: Contributed to conception, design, data acquisition and interpretation, critically revised the manuscript. Ze Lu: Contributed to conception, design, data acquisition and interpretation, performed all statistical analyses, drafted and revised the manuscript. Ruoyan Cao: Contributed to bioinformatics analysis and revised the bioinformatics section of the manuscript. All authors gave their final approval and agreed to be accountable for all aspects of the work.

## FUNDING INFORMATION

This work was supported by the National Natural Science Foundation of China (82370975) and (82170969).

## CONFLICT OF INTEREST STATEMENT

The authors declare no potential conflicts of interest.

## Supporting information


**Figure S1.**Bioinformatics analysis. (A) Enrichment analysis by cell type indicated that the co‐expressed genes exhibited associations with various epithelial cell types, such as tongue epithelial cells and lung epithelial cells. (B) Comprehensive overview of the GO analysis results. (C) Analysis of transcription factors indicated that co‐expressed genes were strongly linked to the transcription factors associated with inflammatory pathways. (D) The analysis of tumour grade showed that lower ZFP36 expression was associated with higher tumour grade.

**Figure S2.**Immunohistochemistry and immunofluorescence. (A) HE staining and immunohistochemical staining results of MCP‐1 proteins in each group of periodontitis and OSCC samples. The results of MCP‐1 staining showed that the expression of MCP‐1 in tissues increased gradually with the aggravation of inflammation.

**Data S1.** Supporting Information

## Data Availability

The data that support the findings of this study are available from the corresponding author upon reasonable request.
