# Supplementary figures and images for: Persistent infection with Porphyromonas gingivalis increases the tumorigenic potential of human immortalised oral epithelial cells through ZFP36 inhibition
Source: Cell Prolif. 2024 Feb 13;57(6):e13609. doi: 10.1111/cpr.13609 (PMC11150143; doi:10.1111/cpr.13609)

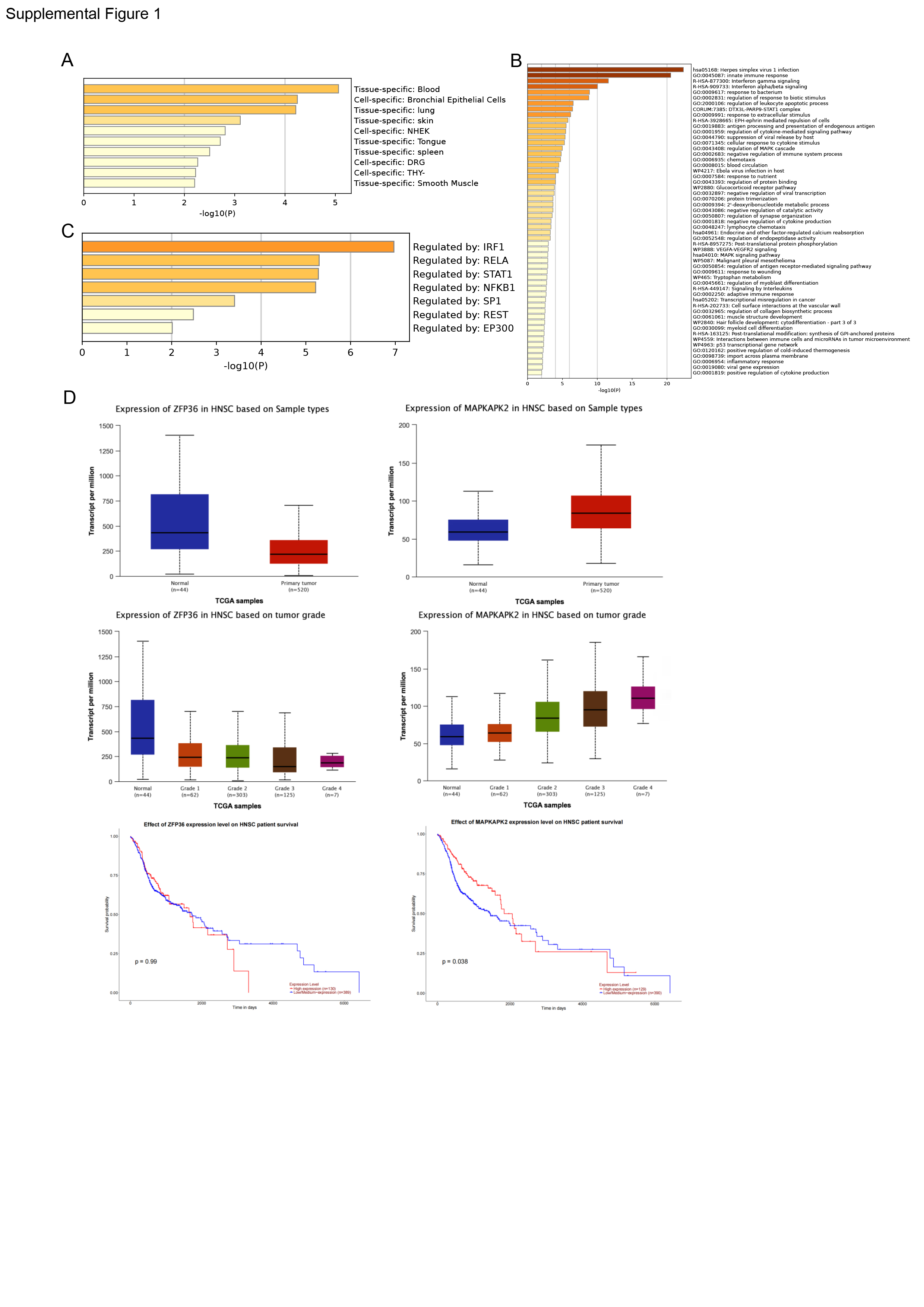

Supplement: Supplementary file 1 — Figure S1.Bioinformatics analysis. (A) Enrichment analysis by cell type indicated that the co‐expressed genes exhibited associations with various epithelial cell types, such as tongue epithelial cells and lung epithelial cells. (B) Comprehensive overview of the GO analysis results. (C) Analysis of transcription factors indicated that co‐expressed genes were strongly linked to the transcription factors associated with inflammatory pathways. (D) The analysis of tumour grade showed that lower ZFP36 expression was associated with higher tumour grade. [file CPR-57-e13609-s002.jpg]

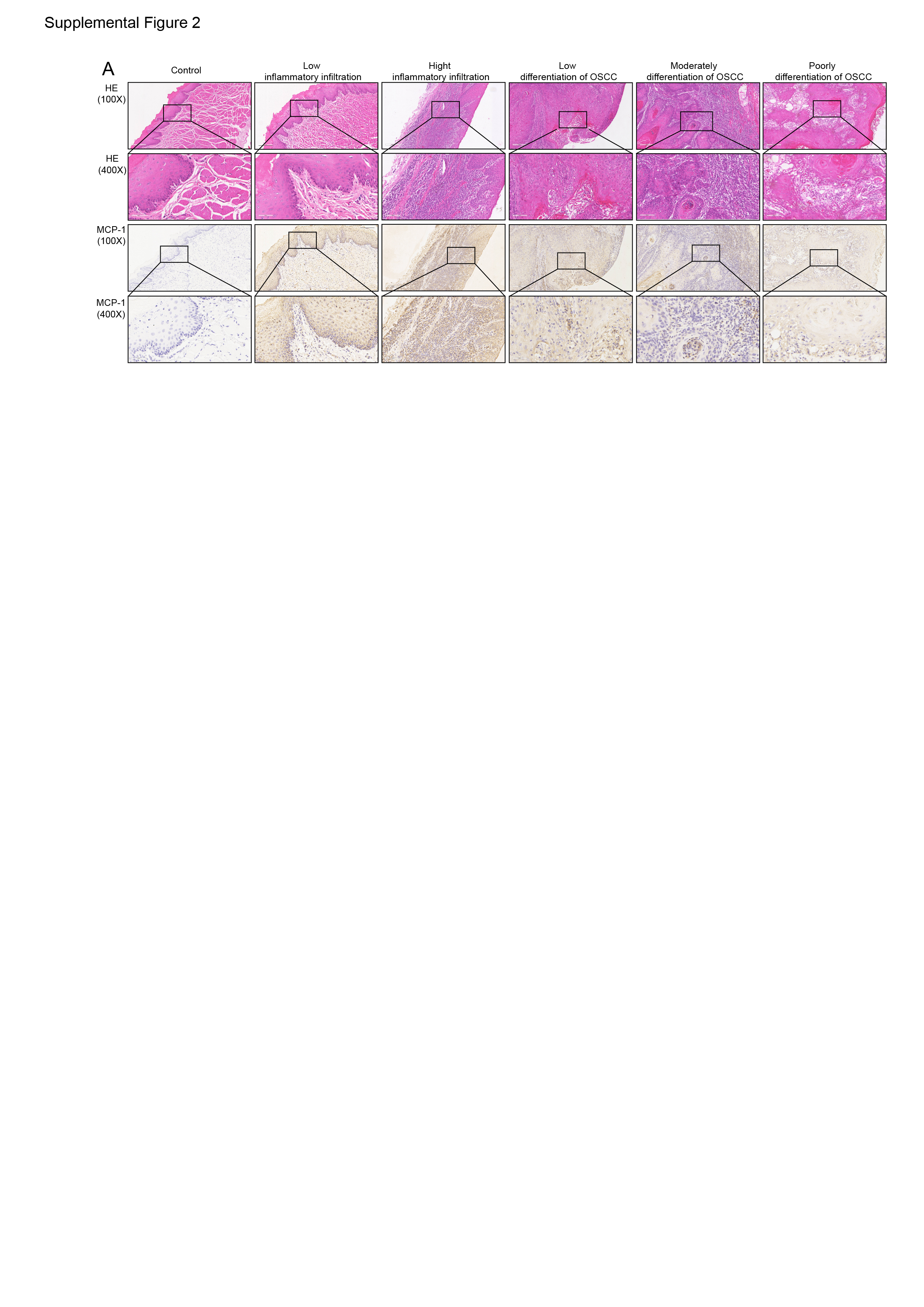

Supplement: Supplementary file 2 — Figure S2.Immunohistochemistry and immunofluorescence. (A) HE staining and immunohistochemical staining results of MCP‐1 proteins in each group of periodontitis and OSCC samples. The results of MCP‐1 staining showed that the expression of MCP‐1 in tissues increased gradually with the aggravation of inflammation. [file CPR-57-e13609-s003.jpg]
