# Supplementary material for: Persistent infection with Porphyromonas gingivalis increases the tumorigenic potential of human immortalised oral epithelial cells through ZFP36 inhibition
Source: Cell Prolif. 2024 Feb 13;57(6):e13609. doi: 10.1111/cpr.13609 (PMC11150143; doi:10.1111/cpr.13609)
Supplement: Supplementary file 3 — Data S1. Supporting Information [file CPR-57-e13609-s001.docx]

**Appendix**

**Persistent infection with *Porphyromonas gingivalis* increases the tumorigenic potential of human immortalized oral epithelial cells through ZFP36 inhibition**

Ze Lu^a^, Ruoyan Cao^a^, Fengxue Geng^a^*, Yaping Pan^a^*

**1 Materials and methods**

**TCGA database analysis**

Data were downloaded from TCGA database, head and neck squamous cell carcinoma regions were selected, and the data were sorted out to obtain the gene expression matrix. The expression levels of target genes (CCAT1, ZFP36, MAPKAPK2 (MK2), E2F1, caspase3, MMP1, MMP2, CDH2, IL1B, IL6, CXCL8) were extracted from the expression matrix, and heat maps were drawn using pheatmap R language package. Ggpubr R language package was used for boxplot drawing, and the difference statistical method was Wilcox test.

**Clinical sample collection**

Tissue samples of oral squamous cell carcinoma were selected from the department of Pathology, Stomatological Hospital affiliated to China Medical University from 2019 to 2021, and pathologically diagnosed as oral squamous cell carcinoma were selected as the positive control group, and were divided into the well-differentiated squamous cell carcinoma group, the moderately differentiated squamous cell carcinoma group, and the poorly differentiated squamous cell carcinoma group. Tissue samples from patients who were clinically diagnosed with chronic periodontitis and needed tooth extraction at the Periodontal Department of Stomatology Hospital affiliated to China Medical University from 2021 to 2022 were selected as the experimental group, which were divided into low inflammatory infiltration group and high inflammatory infiltration group. The gingival tissues of healthy or clinically healthy patients who needed tooth extraction for orthodontic treatment or wisdom tooth extraction were selected as healthy controls. For the above groups, 3 people in each group selected 3 slices. Clinical samples were taken from the size of mung bean grains and stored in the [formaldehyde](D:/QQ%E6%8B%BC%E9%9F%B3%E8%BE%93%E5%85%A5%E6%B3%95/Youdao/Dict/8.10.0.0/resultui/html/index.html#/javascript:;) [solution](D:/QQ%E6%8B%BC%E9%9F%B3%E8%BE%93%E5%85%A5%E6%B3%95/Youdao/Dict/8.10.0.0/resultui/html/index.html#/javascript:;). Ethical Approval: No. 10, Kelun Shenzi, Stomatological Hospital affiliated to China Medical University, 2021. Ethics Code: K2021010

**Immunohistochemical staining**

Clinical samples were grouped and numbered, embedded in wax blocks, sliced, and baked (60℃ for 1 hours). Dewaxing, rehydration (xylene, gradient ethanol, distilled water, hydrogen peroxide), microwave repair (0.01M citric acid buffer solution, microwave 98℃-100℃) repeated twice. The sections were cooled naturally to room temperature and washed by PBS 3 times, 5 minutes each. Sealed tablet (5% BSA, 20 minutes at room temperature). Primary antibody ZFP36 (Cat. no. Sc-374305; Santa Cruz) and MK2 (Cat. No. NBP2-56233; Novus Biologicals) was incubated (37℃, 1 hours, or 4℃ overnight). Wash with PBS for 3 times, 3 minutes each. Second antibody was incubated (37℃, 15-30 minutes). Wash with PBS for 3 times, 3 minutes each. Add SABC drops (37℃, 30 minutes). Wash with PBS for 3 times, 5 minutes each. Add color developing agent to 1ml distilled water and mix well. DBA chromogenic agent (Gene Tech, Shanghai, China) was added to slices, and the reaction time was detected under microscope (about 5 minutes) at room temperature with distilled water. Hematoxylin was redyed for 2 minutes and washed with distilled water. Ethanol gradient dehydration, gum sealing, microscopic examination.

**Cell Proliferation (CCK-8)**

Add 100 μl PBS around the 96-well plate to resist evaporation, and add exponential growth cell suspension 100 μl to the other Wells according to the groups, 2000 cells per well, and culture in an incubator for 24 hours adherent (37℃, 5% CO_2_) for 24 hours,48 hours,72 hours,96 hours, respectively. Add 10μl CCK-8 detection solution (Dojindo, Beijing, China) to each well, and incubate for 2 hours away from light. The absorbance at 450nm was detected.

**Cell Cycle Analysis**

1-5 × 10^6^ cells were collected in the centrifuge tube and washed with cold PBS twice, 5 mintues each. 500ul PBS was resuspended, cold ethanol was slowly added to 75%, and fixed at -20℃ for 60 mintues or -4℃ overnight (12-16h). Centrifuge 1000g for 10 minutes, discard supernatant. Cells were washed by cold PBS at 1000g for 10 mintues. 500ul cold PBS suspension. Add 20ul RnaseA solution (meilunbio, No.MA0334, Dalian, China) and take a water bath at 37℃ for 30 minutes. Centrifuge 1000g for 10 mintues, discard supernatant. Add 400ul PI dye (meilunbio, No.MA0334, Dalian, China) and suspend again. After blending, avoid light at 4℃ for 60mintues. Computer detection (excitation wavelength 488nm)

**Cell** [**apoptosis**](D:/QQ%E6%8B%BC%E9%9F%B3%E8%BE%93%E5%85%A5%E6%B3%95/Youdao/Dict/8.9.8.0/resultui/html/index.html#/javascript:;) **Analysis**

Cells were inoculated in 6-well plates during exponential growth period, and 3 more holes were made for FITC PI staining and NC blank control. The cells were digested by trypsin without EDTA into the centrifuge tube. Cold PBS was suspended twice, and PBS was discarded. Cells were suspended with 400ul Annexin V binding solution for each sample. Annexin V-FITC (KeyGEN, Cat number: KGA105-KGA108, Nanjing, China) 5 μl staining solution was added to each sample and mixed. The samples were incubated at 2-8℃ for 15 mintues under dark conditions. Each sample was mixed with 10ul Propidium Iodide (PI) staining solution (KeyGEN, Cat number: KGA105-KGA108, Nanjing, China) and incubated at 2-8℃ for 5 mintues under dark conditions.

**Cell Migration (Wound Healing ) experiments**

Mark evenly (0.5-1cm) behind 6-hole plate with marker and cross the hole. About 5 × 10^5^ cells were added into the well, and the degree of cell to confluence was above 90%. The cells were pretreated with mitomycin (1μg/ mL) for 1h to inhibit cell division. Line the back with the 1ml tip. The cells were washed with PBS for 3 times, and the cells were removed. Culture in a 37℃ 5% CO_2_ incubator. Samples were taken at 0 hours and 48 hours time points and photos were taken under the microscope. The healing rate was analyzed.

**Cell invasion Assays**

Dissolve the base glue at 4℃ overnight. The centrifuge tube and nozzle were pre-cooled at -20℃ before the test. Matrigel gel (Corning Matrigel Basement Membrance Matrix, Two Oak Park, Bedford) was diluted with serum-free cold cell medium DMEM on ice. Take 100 ul of diluted glue and add to 24-well transwell upper chamber. Transwell was incubated at 37℃ for at least 4 hours. Wash gel lightly in serum-free medium. The serum-free medium was used to suspend the index growing cells (5 × 10^5^cells/ml, 200 ul) and added into the upper chamber. 600 ul cell culture medium containing 10% FBS was added into the lower chamber. 37℃, 24 hours. A cotton swab wipes away the cells in the upper compartment. Remove the transwells, invert and air dry. Add 500 μl 0.1% crystal violet solution, place the chamber in it, take it out at 37℃ for 30 minutes, and wash with PBS. Taking pictures under a microscope, counting.

**Colony formation experiments**

Cells at exponential growth stage were taken and cell suspension was prepared by conventional digestion and passage. The cell suspension was repeatedly blown to disperse the cells fully, the cells were counted, and the cell concentration was adjusted by medium. Add 500 cells to a 6-well plate and gently shake the dish in a cross direction to disperse the cells evenly. Culture at 37℃ in 5% CO_2_ for 2 weeks, and replace fresh culture medium timely according to the PH change of culture medium. When visible clones appeared in the petri dish, the culture was terminated, the culture medium was discarded, and PBS solution was carefully immersed twice. Methanol was fixed for 15 minutes, and air was dried after methanol was discarded. Dye with Giemsa solution (Solarbio, Beijing, China) for 10 minutes, wash away the solution slowly with running water, dry with air and count.

**Quantitative Real-time Polymerase Chain Reaction**

Total RNA was extracted from tissue or cultured cells using TRIZOL reagent (Invitrogen, Carlsbad, CA, USA). Qrt-pcr uses a reverse transcription kit (Takara, Dalian, China) to reverse transcribe RNA into cDNA. SYBR Green (Takara, Dalian, China) was used for real-time PCR analysis. Primer sequences are shown in the table 1 below.

**Appendix Table 1 Primer sequences**

| **Gene name** | **Sequence (5' -> 3')** |
| --- | --- |
| CCAT1-R | TGAATTGAACCTGGACTATGAGAG |
| CCAT1-F | CTGGAAGTAGCACGGAAGAAG |
| ZFP36-R | TGAATTGAACCTGGACTATGAGAG |
| ZFP36-F | CTGGAAGTAGCACGGAAGAAG |
| MAPKAPK2-R | GGGCGAATTTCTCCTGGGTC |
| MAPKAPK2-F | CGCAGTTCCACGTCAAGTC |
| E2F1-R | TCCTGGGTCAACCCCTCAAG |
| E2F1-F | ACGCTATGAGACCTCACTGAA |
| caspase3-R | CTGTACCAGACCGAGATGTCA |
| caspase3-F | CATGGAAGCGAATCAATGGACT |
| MMP1-R | CATGGAAGCGAATCAATGGACT |
| MMP1-F | AAAATTACACGCCAGATTTGCC |
| MMP2-R | GGTCACATCGCTCCAGACT |
| MMP2-F | TACAGGATCATTGGCTACACACC |
| CDH2-R | ATGCACATCCTTCGATAAGACTG |
| CDH2-F | TCAGGCGTCTGTAGAGGCTT |
| IL1B-R | GTCGGAGATTCGTAGCTGGA |
| IL1B-F | ATGATGGCTTATTACAGTGGCAA |
| CXCL8-R | AACCCTCTGCACCCAGTTTTC |
| CXCL8-F | TTTTGCCAAGGAGTGCTAAAGA |

**Western Blotting**

Cell protein lysates were isolated with 10% sodium dodecyl, transferred to 0.22 M NC membrane (Sigma) by sulfate-polyacrylamide gel electrophoresis (SDS-PAGE), and incubated with specific antibodies . The automatic gamma-ray map is made of density meter (Quantity One software; Bio-Rad). GAPDH was used as the control. ZFP36, MK2, GAPDH antibodies from Abcam (Hong Kong, China). MK2 antibody (Cat. No. NBP2-56233; Novus Biologicals)

**Plasmid Transfection**

Inhibition and overexpression plasmids of CCAT1 and ZFP36 were constructed by Genechem cooperation (Shanghai, China) and extracted according to the specification of Rapid Mini Plasmid Kit (Biomad, Beijing, China). Lipo2000 was transfected with active growth cells with a convergence rate of 60% for 24 hours. Normal cultured cells, no-load cells and lipo2000 cells were used as controls. The transfection efficiency was detected by RT-PCR and (or) Western blot. The expression of related genes and proteins were detected after transfection.

**Statistical Analysis**

ONE-WAY ANOVA-LSD multiple comparison method or rank sum test was used for statistical analysis. The level of detection was double-sided α=0.05. All the experiments were repeated for at least three times. All statistical results were performed using the statistical analysis software IBM SPSS Statistics version 24. *P*<0.05 was considered statistically significant.

**Supplemental Figure Legend**

**Supplemental Figure 1. Bioinformatics analysis. A** Enrichment analysis by cell type indicated that the co-expressed genes exhibited associations with various epithelial cell types, such as tongue epithelial cells and lung epithelial cells. **B** Comprehensive overview of the GO analysis results. **C** Analysis of transcription factors indicated that co-expressed genes were strongly linked to the transcription factors associated with inflammatory pathways. **D** The analysis of tumor grade showed that lower ZFP36 expression was associated with higher tumor grade.

**Supplemental Figure 2. Immunohistochemistry and immunofluorescence. A** HE staining and immunohistochemical staining results of MCP-1 proteins in each group of periodontitis and OSCC samples. The results of MCP-1 staining showed that the expression of MCP-1 in tissues increased gradually with the aggravation of inflammation.
